# Evaluation of large language models for antimicrobial classification: implications for antimicrobial stewardship programs

**DOI:** 10.1017/ash.2025.10235

**Published:** 2025-12-01

**Authors:** Tan Vo, Kushal Dahal, Michael Klepser, Benjamin Pontefract, Kaylee E. Caniff, Minji Sohn

**Affiliations:** 1 College of Pharmacy, Ferris State University, 220 Ferris Dr., Big Rapids, MI, 49307, USA; 2 Collaborative to Advance Pharmacy Enterprises (CAPE), Grand Rapids, MI, USA

## Abstract

**Objective:**

To evaluate the ability of large language models (LLMs) with targeted feedback to classify medications as antimicrobial or non-antimicrobial and their implication in antimicrobial stewardship.

**Design:**

Cross-sectional evaluation using a two-phase process: initial unguided classification and feedback-informed reclassification.

**Setting:**

Medication-level analysis of health system prescribing data.

**Participants:**

A data set of 7,239 unique medication entries from health systems in the Collaboration to Harmonize Antimicrobial Registry Measures (CHARM) project.

**Methods:**

Four LLMs, including ChatGPT-3.5, Copilot GPT-4o, Claude Sonnet 4, and Gemini 2.5 Flash, classified all entries against a manual reference standard. Models then received feedback on 20% of misclassified cases for reclassification. Metrics included accuracy, macro F1-score (95% confidence intervals via bootstrap resampling), positive predictive value, negative predictive value, processing time, and error reduction rates (ERRs). McNemar’s test assessed accuracy between phases and model differences.

**Results:**

Baseline accuracy varied among LLMs. Postfeedback, all accuracies improved significantly (*P* < .001) to Gemini (99.6%), Claude Sonnet 4 (99.4%), ChatGPT-3.5 (81.0%), and Copilot (79.7%). Gemini achieved the highest macro-F1 (98.9%, 95% CI: 98.4–99.3) and ERR (69.2%). Processing time were fastest for Copilot (42 s), followed by ChatGPT-3.5 (47 s), Gemini (1,080 s), and Claude (3,475 s). Manual classification of this task is estimated to take 18 hours without LLM. Misclassification was most common among antiseptics, antiparasitics, and drugs with antimicrobial components for non-infectious uses (eg, sulfasalazine).

**Conclusions:**

Top-performing LLMs achieved accuracy levels suitable for automating initial antimicrobial classification in stewardship workflows. Performance variability underscores the need for careful selection and continued human oversight in clinical applications.

**Summary:**

Four LLMs were evaluated for antimicrobial classification using 7,239 medications. Claude Sonnet 4 and Gemini achieved>99% accuracy, while ChatGPT-3.5 and Copilot showed substantial limitations. Top performers could automate stewardship workflows with appropriate oversight.

## Introduction

Antimicrobial stewardship programs are essential for optimizing antimicrobial use, improving patient outcomes, and minimizing resistance.^1^ Clinicians and pharmacists in this area often analyze large datasets containing thousands of medication entries with inconsistent naming conventions, abbreviations, and formatting. A foundational step in such efforts is classifying whether each medication is an antibiotic, antifungal, antiviral, or non-antimicrobial. This classification process can be time-consuming and error-prone when performed manually, especially across diverse healthcare systems with varying documentation practices. Cleaning and standardizing large formularies or multi-site datasets often require extensive manual review of thousands of unique medication entries before stewardship analytics can begin.

Artificial intelligence (AI), particularly large language models (LLMs), may offer a means to streamline this classification step. Unlike traditional rule-based or keyword-matching natural language processing tools, LLMs can interpret abbreviations, misspellings, or brand-generic hybrids through contextual reasoning, enabling automated recognition across diverse data sources.^2,3^ This adaptability makes them uniquely suited for large-scale stewardship databases where variation is the norm. AI has already demonstrated potential in related pharmacy domains: ChatGPT-4.0 accurately solved all 39 medication therapy management cases in one study, successfully identifying interactions and recommending therapy management plans.^4^ In pharmacovigilance, AI tools have identified adverse drug events and reactions (57.6%), processed safety reports (21.2%), extracted drug-drug interactions (7.6%), and guided personalized care through toxicity prediction (7.6%) or side effect identification (3.0%).^5^ These capabilities underscore AI’s strength in processing large-scale data, through most use cases in pharmacy remain exploratory.^5^

Despite the promise, generative models such as ChatGPT-3.5 may underperform on complex pharmacy tasks requiring clinical reasoning.^6–8^ In educational assessments, ChatGPT-3.5, Microsoft Copilot, and Google’s Gemini scored lower than students on calculation and case analyses.^7^ Another evaluation found that ChatGPT-3.5 performed well on basic drug knowledge but occasionally produced incomplete or fabricated information.^8^ Moreover, LLMs can “hallucinate”, confidently generating inaccurate information.^9,10^ Prior studies have cautioned that unsupervised LLMs use could endanger infectious-disease consultation quality. Accordingly, validation and human oversight are mandatory when applying LLMs in stewardship contexts.^10,11^

This study therefore aimed to evaluate the ability of several publicly available LLMs to classify large-scale medication lists as antimicrobial or non-antimicrobial and to assess whether targeted feedback improves performance. Demonstrating high accuracy, high macro-F1 (sensitivity-balanced) and efficient processing would support responsible LLM integration into stewardship workflows.

## Methods

### Study design and setting

This cross-sectional evaluation assessed four publicly available LLMs for classifying medications as antimicrobials or non-antimicrobials. The study followed a two-phase design: (1) initial, “naïve” classification without reference guidance, and (2) reclassification with partial gold-standard feedback.

### Data set and reference standard

Medication entries were extracted from the Collaboration to Harmonize Antimicrobial Registry Measure (CHARM) project database, a Ferris State University initiative aggregating outpatient prescription records from Michigan health systems to assess antibiotic use (see Appendix 1 for CHARM description). Entries reflected unprocessed prescription text exactly as entered in the electronic health records, including brand and generic names, abbreviations, strengths, dosage form, or route of administration (see Appendix 2 for a sample of the data set). After deduplication of identical entries, the final data set contained 7,239 unique medications for classification.

Each entry was manually classified by three PharmD student researchers under the supervision of licensed pharmacist faculty. Disagreements were resolved by group discussion and consensus, using UpToDate Lexidrug and Micromedex. The entire classification process required approximately 18 hours of initial labeling and 5 hours of faculty review, for an estimated total of 23 hours of manual effort.

### Study objectives

The primary objective was to evaluate baseline accuracy of each LLM in distinguishing antimicrobial from non-antimicrobial medications. Secondary objectives were to evaluate accuracy gains following feedback, compare cross-model performance metrics, and identify common misclassification themes.

### Evaluated LLMs

Four general-purpose LLM models were selected based on specific inclusion criteria relevant to practical implementation in clinical and public health settings: freely accessible at the time of study (no subscription or institutional license required), user-friendly (no coding expertise), and capability of automated classification (either by accepting a pasted list of medications or through file upload functionality). The models meeting these criteria were ChatGPT (GPT-3.5; OpenAI), Microsoft Copilot (GPT-4o; Microsoft/OpenAI), Claude Sonnet 4 (Anthropic), and Gemini 2.5 Flash (Google). Platforms were accessed through publicly available web-based chatbot interfaces between June 15 and July 14, 2025. During the study, ChatGPT-3.5 and Copilot supported file upload; Claude Sonnet 4 and Gemini required smaller batches due to input limits (see Table 1 for model key attributes).

**Table 1.** Summary of LLMs evaluated in this study

Model	Developer	Version	Access platform	Input method used	Estimated entries per query
ChatGPT-3.5	OpenAI	GPT-3.5	https://chatgpt.com	File upload (batch-capable)	Full list
Copilot	Microsoft/OpenAI	GPT-4o	Bing Chat via Edge		Full list
Claude	Anthropic	Claude Sonnet 4	https://claude.ai	Pasted text (file upload not viable)	250
Gemini	Google	Gemini 2.5 Flash	https://gemini.google.com		500

### Prompt development and implementation steps

All LLMs received identical prompts. Chat histories, caches, and cookies were cleared prior to phase 1. Phase 2 was conducted in the same conversation thread to preserve contextual continuity between classification rounds.

#### Initial prompt (phase 1 – unguided, naïve classification)

In the first round, no external references or lists were provided. The medication list (file A) was uploaded, and each model received the following prompt:

“You are a clinical pharmacist. Classify each of the following medications into one of these four categories: Antibiotic, Antifungal, Antiviral, or Non-antimicrobial. Present your results in a table format with two columns:

1. Original medication name (exactly as listed)
2. Classification”

#### Second prompt (phase 2- feedback-informed reclassification)

After comparing phase 1 outputs with the reference standard, two columns were appended: class_match (Yes/No) indicating whether the classification agreed with the reference, and correct_class showing the true category.

The table was then filtered to include only misclassified entries (ie, class_match = No) and randomly sampled 20% to create a feedback file (file B) based on pilot testing (representation vs token limits). File B contained two columns: Original Medication Name and correct_class.

The full list (file A) and feedback sample (file B) were uploaded together, and each LLM was instructed to re-evaluate the entire medication list (file A), using file B only as a reference guide. This approach tested each model’s ability to generalize from partial feedback rather than memorize an answer key. The following prompt was used:

“Review your classifications and double-check for accuracy. This is the matching table for reference between standard and your results that are mismatched (“No” for both class_match column) and their correct classification (correct_class column). Pay special attention to: brand names and abbreviations, combination medications (like Augmentin or Paxlovid) - ensure the cleaned name reflects the antimicrobial components, newer antiviral medications, topical vs systemic formulations, medications that are commonly confused with antimicrobials. Please provide a revised table if you identify any corrections needed.”

### Performance evaluation

Outputs were compared with the reference standard for both phases using the full data set to ensure identical sample sizes. Metrics were reported primarily with macro-averaging (equal weight to each class) over weighted-averaging to avoid bias from the large non-antimicrobials) class.

1. Accuracy: (TP+TN)/(TP+TN+FP+FN), proportion of all medications correctly classified.
2. Precision (Positive Predictive Value—PPV): TP/(TP+FP), proportion of predicted positives that were true positives.
3. Negative Predictive Value (NPV): TN/(TN+FN), proportion of predicted negatives that were true negatives.
4. Recall (Sensitivity): TP/(TP+FN), proportion of actual positives that were correctly predicted.
5. Specificity: TN/(TN+FP), proportion of actual negatives that were correctly predicted.
6. F1 Score: 2 × [(Precision×Recall)/(Precision+Recall)], harmonic mean of precision and recall
7. Error Reduction Rate (ERR): (Error phase 2 − Error phase 1)/Error phase 1, percentage decrease in total number of misclassified entries from phase 1 to phase 2
8. Processing Time (in seconds): recorded for phase 1 only, measured from prompt submission to output completion

### Error pattern identification

Misclassification review used the top-performing phase 2 model (based on accuracy and macro-F1). All discordant entries were examined and grouped thematically by pharmacologic class, clinical indication, and naming pattern.

### Statistical analysis

The 95% confidence intervals (95% CIs) were reported for all applicable metrics. Accuracy was estimated with Wilson score 95% CIs, whereas precision, NPV, recall (sensitivity), specificity, and F1 score were calculated with nonparametric bootstrap percentile 95% CIs based on 2,000 resamples stratified by the reference class to account for class imbalance. Within-model phase 1 and phase 2 improvement (four tests, one perm model) and between-model phase 2 comparisons (six pairwise tests) were evaluated using McNemar’s tests with Holm-Bonferroni adjustment for multiple comparisons. Descriptive macro-levels CIs were also reported for interpretability.

Analyses were performed in R (R Core Team, 2025) using the *boot* package for bootstrap and *binom* for Wilson CIs. Tables and figures were prepared in Microsoft Excel (Professional Plus, 2021) with significance defined as α <.05. This study received a waiver of consent as it was deemed not human subjects research by the Ferris State University Institutional Review Board.

## Results

### Baseline model performance (phase 1)

**Figure 1.**
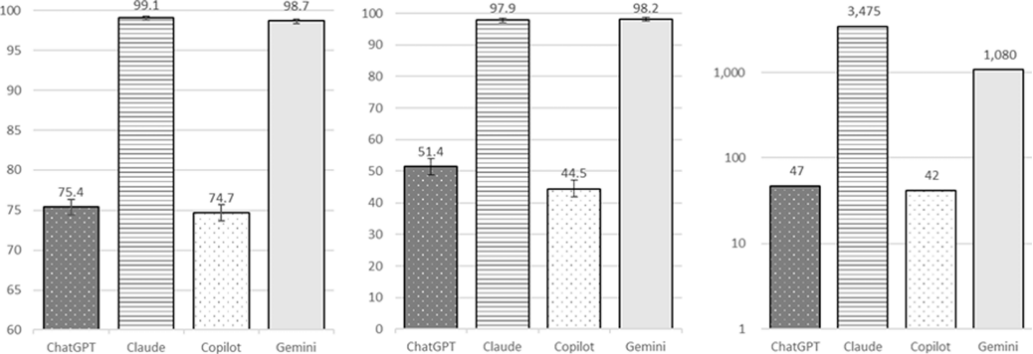
Performance comparison of LLMs in antimicrobial classification: phase 1 (Unguided). Bar charts showing difference in performance metrics among 4 LLMs (ChatGPT-3.5, Claude Sonnet 4, Copilot, and Gemini) in phase 1. Left chart shows accuracy (as percentage and error bar), middle chart shows macro-F1 (as percentage and error bar), and right chart shows processing time (in seconds).

The data set contained 7,239 unique medication entries, consisting of 1,907 (26.4%) antibiotics, 183 (2.5%) antifungals, 280 (3.9%) antivirals, and 4,869 (67.3%) non-antimicrobials. Baseline accuracy varied markedly across models (see Table 2 and Figure 1). Claude Sonnet 4 achieved the highest initial accuracy at 99.1% (95% CI 98.8–99.3), followed closely by Gemini 2.5 Flash at 98.7% (98.4–98.9). In contrast, ChatGPT-3.5 and Copilot (GPT-4o) performed considerably lower, at 75.4% (74.4–76.4) and 74.7% (73.7–75.7), respectively.

**Table 2.** Performance comparison of LLMs in antimicrobial classification: phase 1 (Unguided) vs phase 2 (Feedback-informed)

Model	Phase	Precision (%, 95% CIs)	NPV (%, 95% CIs)	Recall (%, 95% CIs)	Specificity (%, 95% CIs)	Macro-F1 (%, 95% CIs)	Accuracy* (%, 95% CIs)	Processing time (s)	Error reduction rate (%)
ChatGPT-3.5	1	93.3 (93.2–93.4)	93.5 (93.4–93.6)	44.0 (42.0–46.1)	81.2 (80.8–81.7)	51.4 (48.8–54.0)	75.4 (74.4–76.4)	47	–
ChatGPT-3.5	2	94.2 (93.6–94.6)	94.8 (94.6–94.9)	58.5 (56.2–60.9)	85.5 (85.0–86.0)	68.1 (65.8–70.3)	81.0 (80.1–81.9)	–	23.2
Claude Sonnet 4	1	99.5 (99.4–99.6)	99.6 (99.5–99.7)	96.5 (95.3–97.5)	99.4 (99.3–99.6)	97.9 (97.2–98.5)	99.1 (98.8–99.3)	3,475	–
Claude Sonnet 4	2	99.5 (99.1–99.7)	99.7 (99.6–99.8)	8.2 (97.3–99.0)	99.7 (99.6–99.8)	98.8 (98.3–99.3)	99.4 (99.2–99.6)	–	33.3
Copilot	1	93.2 (93.1–93.3)	93.3 (93.3–93.4)	39.1 (37.4–40.9)	80.7 (80.3–81.1)	44.5 (41.9–47.0)	74.7 (73.7–75.7)	42	–
Copilot	2	94.2 (94.1–94.4)	94.5 (94.3–94.6)	48.8 (46.8–51.0)	84.5 (84.1–85.0)	57.4 (54.8–60.1)	79.7 (78.8–80.7)	–	20.0
Gemini	1	97.8 (96.9–98.6)	99.4 (99.3–99.5)	98.6 (98.1–99.0)	99.1 (99.0–99.3)	98.2 (97.6–98.7)	98.7 (98.4–98.9)	1,080	–
Gemini	2	98.1 (97.1–98.8)	99.7 (99.6–99.8)	99.8 (99.8–99.9)	99.9 (99.8–99.9)	98.9 (98.4–99.3)	99.6 (99.4–99.7)	–	69.2

*All models demonstrated statistically significant improvements in accuracy from phase 1 to phase 2 (McNemar’s test, Holm-Bonferroni-adjusted *p* < .05). Exact *p*-values were<001 for ChatGPT-3.5, Copilot, and Gemini, and .009 for Claude Sonnet 4.

Performance ranking by macro-F1 score, which balances precision (positive predictive value, PPV) and recall (sensitivity), again highlighted Claude Sonnet 4 and Gemini as superior to ChatGPT-3.5 and Copilot. Gemini achieved the highest macro-F1 (98.2%, 95% CI 97.6–98.7) with 97.8% precision, 99.4% NPV, and 98.6% recall. Claude followed closely (F1 97.9%, 95% CI 97.2–98.5). ChatGPT and Copilot demonstrated limited recall (44.0% and 39.1%, respectively) despite high precision (93.3 and 93.2%, respectively), yielding macro-F1 scores of 51.4% and 44.5%.

Processing times reflected a trade-off between speed and accuracy. ChatGPT-3.5 and Copilot completed classification in 47 and 42 s respectively via file upload. Gemini processed the data set in 1,080 s (18 mins, batch size 500), while Claude Sonnet 4 required 3,475 s (approximately 58 mins, batch size 250), necessitating approximately 29 separate interactions. For context, manual classification of the same data set by three PharmD students with faculty supervision was extrapolated to 23 hours.

### Impact of feedback-informed classification (phase 2)

**Figure 2.**
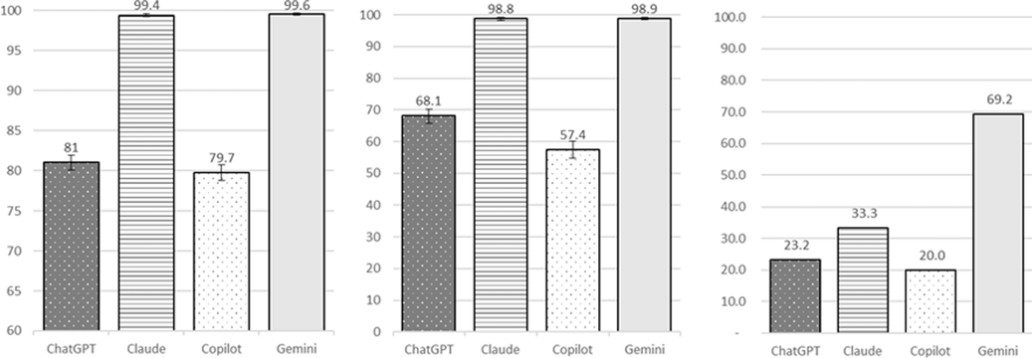
Performance comparison of LLMs in antimicrobial classification: phase 2 (Feedback-informed). Bar charts showing difference in performance metrics among 4 LLMs (ChatGPT-3.5, Claude Sonnet 4, Copilot, and Gemini) in phase 2. Left chart shows accuracy (as percentage and error bar), middle chart shows macro-F1 (as percentage and error bar), and right chart shows error reduction rate (as percentage).

All models demonstrated statistically significant accuracy improvements after feedback (McNemar’s test, Holm-Bonferroni-adjusted *p* < .001 for ChatGPT-3.5, Copilot, and Gemini; *p* = .009 for Claude Sonnet 4; see Table 2 and Figure 2). Gemini achieved the highest phase 2 accuracy (99.6%, 95% CI 99.4–99.7), followed by Claude (99.4%, 95% CI 99.2–99.6). ChatGPT-3.5 accuracy improved to 81.0% (80.1–81.9), and Copilot improved to 79.7% (78.8–80.7).

Evaluation of macro-F1 showed a similar pattern to phase 1. Gemini achieved the highest macro-F1 (98.9%, 95% CI 98.4–99.3), supported by 98.1% precision, 99.7% NPV, and 99.8% recall. Claude Sonnet 4 achieved a macro-F1 of 98.8% (98.3–99.3), supported by 99.5% precision, 99.7% NPV, and 96.5% recall. ChatGPT-3.5 improved its macro-F1 of 68.1% (65.8–70.3), supported by 94.2% precision and 58.5% recall, while Copilot showed the lowest macro-F1 at 57.4% (54.8–60.1), with 94.2% precision and 48.8% recall.

Error reduction rates further illustrated the variable effect of feedback. Gemini showed the largest improvement, reducing misclassification errors by 69.2% despite only 20% of phase 1 errors provided for feedback. Claude Sonnet 4 reduced errors by 33.3, while ChatGPT-3.5 and Copilot showed smaller error reductions of 23.2 and 20.0%, respectively.

To exemplify how targeted feedback improved model performance, Gemini initially misclassified albendazole as an antibiotic and methenamine as non-antimicrobial in phase 1. After receiving partial corrective feedback (albendazole was included in the 20% feedback sample, whereas methenamine was not), Gemini accurately reclassified albendazole as non-antimicrobial and methenamine as an antibiotic in phase 2.

### Comparative model performance

Pairwise comparisons of phase 2 accuracy (see Table 3) found Gemini and Claude Sonnet 4 not significantly different (*χ* ^2^ = 1.7, *P* = .193). Both Gemini and Claude significantly outperformed ChatGPT-3.5 and Copilot (all *P* < .001). ChatGPT-3.5 demonstrated statistically superior accuracy compared to Copilot (*χ* ^2^ = 10.4, *p* < .01). Overall, only Gemini and Claude reached accuracy and macro-F1 suitable for automated antimicrobial classification.

**Table 3.** Pairwise comparison of LLMs in antimicrobial classification accuracy: phase 2 (Feedback-informed)

Model A	Model B	A wrong, B right	A right, B wrong	χ^2^ (McNemar)	Unadjusted *p*-value	Holm-Bonferroni-adjusted *p*-value
ChatGPT-3.5	Copilot	362	455	10.36	.0013	.0026
ChatGPT-3.5	Claude Sonnet 4	1,355	22	1 288.47	<.001	<.001
ChatGPT-3.5	Gemini	1,371	27	1 290.16	<.001	<.001
Copilot	Claude Sonnet 4	1,448	22	1 381.38	<.001	<.001
Copilot	Gemini	1,464	27	1 383.03	<.001	<.001
Claude Sonnet 4	Gemini	35	24	1.70	.193	.193

### Error pattern analysis

Error pattern analysis was performed using Gemini, the top-performing model in phase 2. Confusion matrix review identified 27 total misclassifications (see Table 4; other model confusion matrix data available in Supplemental Table 9). The errors included antiparasitic medications (amebicides, anthelminthics) incorrectly classified as antibiotics (34.5%, *n* = 10), vaccines (eg, rotavirus, recombinant zoster vaccine) incorrectly classified as antimicrobials based on the microbe they aim to prevent (24.1%, *n* = 7), anti-infective agents with antimicrobial-sounding names (eg, boric acid, podofilox, oxyquinoline) labeled as antivirals or antifungals (24.1%, *n* = 7), and non-infectious medications containing sulfonamide structures (eg, sulfasalazine for inflammatory bowel disease) incorrectly classified as antibiotics (17.2%, *n* = 5).

**Table 4.** Confusion matrix for best-performing LLM (Gemini) in antimicrobial classification: phase 2 (Feedback-informed)

	Predicted antibiotic	Predicted antifungal	Predicted antiviral	Predicted Non-antimicrobial	Total
Actual antibiotic	1,905	2	–	–	1,907
Actual antifungal	–	183	–	–	183
Actual antiviral	–	–	280	–	280
Actual non-antimicrobial	12	5	10	4,842	4,869

### Result table formatting

Formatting outputs varied notably across models. ChatGPT-3.5 and Copilot were the most user-friendly, supporting direct file export (eg, CSV or Excel) with minimal postprocessing. In contrast, Claude Sonnet 4 and Gemini 2.5 Flash generated more restrictive outputs. Claude produced inline text tables that lacked export functionality, and Gemini faced the added limitation of line shifting, where rows occasionally misaligned during export, making consistent table reconstruction more time-consuming.

## Discussion

To our knowledge, this study represents the first systematic evaluation of LLMs for antimicrobial classification using real-world clinical datasets. The findings demonstrate substantial heterogeneity among models: Claude Sonnet 4 and Gemini 2.5 Flash achieved remarkably high accuracy and macro-F1, making them potential tools for fundamental steps of antimicrobial stewardship workflow, whereas ChatGPT-3.5 and Copilot (GPT-4o) remained less reliable even after feedback. ChatGPT-3.5 and Copilot revealed significant limitations in antimicrobial detection, missing nearly half of true antimicrobials (low recall [sensitivity] despite high precision [PPV]), limiting suitability for stewardship applications where comprehensive detection is essential. Such discrepancies emphasizes that LLM competence is not interchangeable across platforms or versions, and that routine benchmarking and validation are essential whenever models are updated or integrated into applied workflows.

The consistent accuracy improvement following feedback demonstrates that LLMs can integrate corrective guidance without retraining, mirroring adaptive reasoning. Despite receiving corrections for only 20% of phase 1 errors, Gemini reduced misclassifications by nearly 70% and Claude Sonnet 4 by one-third, suggesting that targeted feedback loops could optimize accuracy in real-world settings where exhaustive manual review is infeasible.

Processing efficiency varied drastically across models, revealing important workflow trade-offs. ChatGPT-3.5 and Copilot’s processed all entries within one minute via file upload, whereas Claude Sonnet 4 and Gemini required up to an hour due to smaller input windows and manual pasting. Despite these constraints, the trade-off between processing speed and accuracy was remarkable; Claude’s 58-minute processing time yielded 99.1% accuracy, compared with ChatGPT-3.5’s 47-s processing time at only 75.4% accuracy. Even the slowest LLM workflows completed the task in under one hour, compared with approximately 23 hours of manual students and pharmacist review.

However, it is important to note that all tools were public chat interfaces. Therefore, these models are not compliant with the Health Insurance Portability and Accountability Act (HIPAA) and must never be used with identifiable patient data unless deployed within a secure, institutionally hosted server environment. All analyses in this study were limited to de-identified medication names to ensure data privacy and regulatory compliance. Future studies should assess cost-effectiveness compared to manual review, user acceptance and workflow integration of these tools into existing analytic pipelines, including locally hosted or API-based (Application Programing Interface) implementations that preserve data privacy. Exploration of medical-domain LLMs (eg, Med-Gemini) may clarify whether domain-specific pretraining offers consistent advantages.^12^

Misclassification patterns reveal consistent reasoning gaps. Common errors involved medications with antimicrobial-sounding names but non-antimicrobial mechanism antiseptics, antiparasitic, medications containing antimicrobial components for non-infectious indications. These errors may reflect surface-level pattern recognition—for example, reliance on naming cues or contextual associations—rather than full understanding of pharmacologic mechanisms. Changes to the language used in the text prompts could have improved some of these errors, such as specifying that antiparasitic agents and vaccines should not be classified as antimicrobials. Limited confusion between antifungal agents and newer antivirals likely reflects training-data constraints and outdated model cutoffs, as most systems were trained on data preceding 2024.^13^

High-performing LLMs can streamline early, low-risk data-centric tasks in stewardship programs, allowing teams to focus on interpretation rather than data cleaning. Rapid classification could enable automated antimicrobial surveillance dashboards, near-real-time monitoring, and more consistent data across sites. Future applications could extend to standardizing antimicrobial nomenclature, parsing prescription frequencies and durations, and converting free-text into structured data fields, ultimately improving analytic quality and reproducibility.

Model performance represents a temporal snapshot; ongoing updates, training-data changes, and prompt sensitivities can substantially alter result. These findings therefore reflect zero-shot capabilities rather than fixed superiority. As illustrated by newer versions such as ChatGPT-5 and Claude Sonnet 4.5 during the manuscript preparation, LLM performance and scope evolve rapidly.^14,15^ Strong results in one domain (eg, antimicrobial classification) may not necessarily generalize to others, underscoring the need for ongoing validation, transparent version reporting, and reproducible benchmarking.

Several limitations warrant consideration for this project. Our reference standard involved manual classification that may contain subjectivity for edge cases. The study used publicly available chatbot, which prompted HIPAA-concerns. The feedback mechanism was random and limited to 20% of misclassified entries and may not represent maximum achievable improvement. Also, rapid LLM development means our evaluation represents a snapshot for a specific task and may not represent the overall capabilities with future model updates.

In sum, LLMs already demonstrate high accuracy and efficiency for structured antimicrobial classification, a foundational but crucial step for stewardship analytics. Their responsible use requires not only technical validation but also safeguards for privacy, reproducibility, and interpretability, ensuring these tools augment rather than replace human clinical judgment.

## Supporting information

10.1017/ash.2025.10235.sm001 Vo et al. supplementary material 1 Vo et al. supplementary material

10.1017/ash.2025.10235.sm002 Vo et al. supplementary material 2 Vo et al. supplementary material

## Supplementary material

The supplementary material for this article can be found at https://doi.org/10.1017/ash.2025.10235.

## Manuscript preparation

The manuscript was prepared solely by the listed authors. No individuals or organizations outside the author group had any role in the data collection, analysis, or writing of this manuscript.
