# Supplementary material for: Evaluation of large language models for antimicrobial classification: implications for antimicrobial stewardship programs
Source: Antimicrob Steward Healthc Epidemiol. 2025 Dec 1;5(1):e324. doi: 10.1017/ash.2025.10235 (PMC12722537; doi:10.1017/ash.2025.10235)
Supplement: Vo et al. supplementary material 1 — Vo et al. supplementary material [file S2732494X25102350sup001.docx]

**Appendix 1. Collaboration to Harmonize Antimicrobial Registry Measure (CHARM) Description**

**What is CHARM?**

The Collaboration to Harmonize Antimicrobial Registry Measures (CHARM) project is a program developed by faculty at Ferris State University in Big Rapids, Michigan to help quantify and assess appropriateness of outpatient antibiotic use. The CHARM project has been approved by the Ferris State University Institutional Review Board (IRB). CHARM is designed to collect and report outpatient antimicrobial prescribing in clinics, urgent care centers, emergency department, and dental practices. Data are transformed into interactive, user-friendly dashboards that are used by partner institutions to track and report outpatient antimicrobial use. Each CHARM dashboard is equipped with filters that allow for viewing the data according to timeframe, diagnosis, prescriber group/individual prescriber, antimicrobial, guideline concordance, and patient demographics. These filters can be stacked on top of each other to allow significant manipulation of data that can would otherwise be difficult for clinicians who are unfamiliar with working with large data sets.

**Data Acquisition:**

CHARM partners with health systems with outpatient clinics, urgent care centers, emergency department, and dental practices. Under a data use agreement, health-systems securely share de-identified, masked data extracted from their electronic medical records with the CHARM project. These data are used to generate a health-system specific dashboard, which is then made available to clinicians within each health-system. New data is transferred to CHARM on a monthly or quarterly basis to update the dashboards in a timely manner. Partnering health-systems use their personal dashboards to identify and track outpatient antimicrobial use, identify stewardship needs and track the progress of interventions. Using aggregate data from all partnering health-systems, CHARM publishes a statewide dashboard for Michigan and a national dashboard. These aggregate dashboards can be used by partnering health-systems for benchmarking purposes.

**Appendix 2. Sample Medication Entries**

This appendix provides a representative sample of the raw, unprocessed medication entries used in the dataset.

| rx_name |
| --- |
| AMLODIPINE 2.5 MG TABLET |
| amikacin liposomal-neb.accessr 590 mg/8.4 mL NbSp |
| AMLODIPINE/VALSARTAN/HCTHIAZID |
| AMOXICILLIN-POT CLAVULANATE 200-28.5 MG ORAL CHEW |
| AMOXICILLIN-POT CLAVULANATE 250-62.5 MG/5 ML ORAL SUSR |
| Azithromycin 200 MG/5ML Suspension Reconstituted |
| bacitracin (MYCITRACIN) 500 unit/gram ointment |
| CARBAMIDE PEROXIDE 6.5 % EAR DROPS |
| cefepime 1 gram SolR |
| CEFTAZIDIME IV |
| Chloroquine Phosphate 500 MG Tablet |
| clindamycin (CLEOCIN) 600 mg/50 mL IVPB |
| CLINDAMYCIN 75 MG/5 ML ORAL SOLUTION |
| CLOBAZAM |
| COLLAGENASE CLOSTRIDIUM HIST. |
| dalbavancin HCl (DALVANCE IV) |
| demeclocycline |
| DOXYCYCLINE HYCLATE 50 MG ORAL TBEC |
| doxycycline hyclate 50 mg Tab |
| EFLORNITHINE 13.9 % TOPICAL CREAM |
| ENSURE ORIGINAL ORAL LIQUID |
| FILGRASTIM-SNDZ 480 MCG/0.8 ML INJECTION SYRINGE |
| gentamicin-prednisoLONE (PRED-G) 0.3-1 % ophthalmic drops |
| GLYBURIDE 2.5 MG TABLET |
| GLYBURIDE 5 MG TABLET |
| Hydrocortisone-Acetic Acid |
| IODOFORM |
| L. ACIDOPHILUS/L. RHAMNOSUS |
| LINACLOTIDE |
| MENING C,Y,W-135 VAC 1 OF 2/PF |
| MENTHOL |
| METRONIDAZOLE 0.75 % VAGINAL GEL |
| MONTELUKAST 5 MG CHEWABLE TABLET |
| MULTIVITAMIN WITH IRON |
| MULTIVITAMIN WITH MINERALS |
| NITROFURANTOIN MONO-MCR 100 MG |
| NOVOLIN 70-30 FLEXPEN U-100 INSULIN 100 UNIT/ML (70-30) SUBCUTANEOUS |
| nystatin |
| OMEPRAZOLE 2 MG/ML ORAL SUSPENSION |
| PAROXETINE MESYLATE (MENOPAUSAL SYMPTOMS SUPPRESSANT) 7.5 MG CAPSULE |
| POTASSIUM CHLORIDE ER 20 MEQ TABLET,EXTENDED RELEASE |
| PREDNISOLONE SOD PH/BROMF/PF |
| PSYLLIUM HUSK |
| RISPERIDONE 3 MG TABLET |
| RITALIN 5 MG TABLET |
| RIVAROXABAN 20 MG TABLET |
| SYRINGE WITH NEEDLE, 5 ML |
| TRETINOIN MICROSPHERES |
| trimethoprim (TRIMPEX) 100 mg tablet |
| vancomycin 125 mg/2.5 mL Syrg |

**Appendix 3. Performance metric formulas**

The following formulas were used to evaluate model performance:

Let:

- TP = True Positives
- FP = False Positives
- TN = True Negatives
- FN = False Negatives
- N = Total number of entries

1. Accuracy = (TP + TN) / N
2. Precision (Positive Predictive Value) = TP / (TP + FP)
3. Recall (Sensitivity) = TP (TP + FN)
4. F1 Score = 2 x (Precision x Recall) / (Precision + Recall)
5. Specificity = TN / (TN + FP)
6. Macro-Average F1 Score = Mean of F1 scores across all classes
7. Error Reduction Rate = (Errors in Phase 1 – Errors in Phase 2) / Errors in Phase 1
