# Supplementary material for: Evaluation of large language models for antimicrobial classification: implications for antimicrobial stewardship programs
Source: Antimicrob Steward Healthc Epidemiol. 2025 Dec 1;5(1):e324. doi: 10.1017/ash.2025.10235 (PMC12722537; doi:10.1017/ash.2025.10235)
Supplement: Vo et al. supplementary material 2 — Vo et al. supplementary material [file S2732494X25102350sup002.docx]

SUPPLEMENTAL TABLES

**Supplemental Table 1**. True positives (TPs), true negatives (TNs), false positives (FPs), and false negatives (FNs) for ChatGPT-3.5 by antimicrobial class: Phase 1 (Unguided) vs Phase 2 (Feedback-informed), n = 7,239 medication entries

| Class | Phase | TPs (n, %) | TNs (n, %) | FPs (n, %) | FNs (n, %) |
| --- | --- | --- | --- | --- | --- |
| Antibiotic | 1 | 474 (6.5) | 5,332 (73.7) | 0 (0) | 1,433 (19.8) |
|  | 2 | 787 (10.9) | 5,332 (73.7) | 0 (0) | 1,120 (15.5) |
| Antifungal | 1 | 51 (0.7) | 7,056 (97.5) | 0 (0) | 132 (1.8) |
|  | 2 | 94 (1.3) | 7,055 (97.5) | 1 (0) | 89 (1.2) |
| Antiviral | 1 | 65 (0.9) | 6,959 (96.1) | 0 (0) | 215 (3.0) |
|  | 2 | 116 (1.6) | 6,959 (96.1) | 0 (0) | 164 (2.3) |
| Non-antimicrobial | 1 | 4,869 (67.3) | 590 (8.2) | 1,780 (24.6) | 0 (0) |
|  | 2 | 4,869 (67.3) | 998 (13.8) | 1,372 (19.0) | 0 (0) |

**Supplemental Table 2.** Performance classification metrics for ChatGPT-3.5 by antimicrobial class: Phase 1 (Unguided) vs Phase 2 (Feedback-informed)

| Class | Phase | Precision  (%, 95% CIs) | NPV  (%, 95% CIs) | Recall  (%, 95% CIs) | Specificity (%, 95% CIs) | F1 Score  (%, 95% CIs) | Accuracy  (%, 95% CIs) |
| --- | --- | --- | --- | --- | --- | --- | --- |
| Antibiotic | 1 | 100  (99.2-100) | 78.8  (77.8-79.8) | 24.9  (23.0-26.8) | 100  (99.9-100) | 39.8  (37.3-42.3) | 80.2  (79.3-81.1) |
|  | 2 | 100  (99.5-100) | 82.6  (81.7-83.5) | 41.3  (39.1-43.5) | 100  (99.9-100) | 58.4  (56.1-60.5) | 84.5  (83.7-85.3) |
| Antifungal | 1 | 100  (93.0-100) | 98.2  (97.8-98.4) | 27.9  (21.9-34.8) | 100  (99.9-100) | 43.6  (35.9-51.2) | 98.2  (97.8-98.5) |
|  | 2 | 98.9  (94.3-99.8) | 98.8  (98.5-99.0) | 51.4  (44.2-58.5) | 100  (99.9-100) | 67.6  (60.8-73.3) | 98.8  (98.5-99.0) |
| Antiviral | 1 | 100  (94.4-100) | 97.0  (96.6-97.4) | 23.2  (18.7-28.5) | 100  (99.9-100) | 37.7  (30.8-44.4) | 97.0  (96.6-97.4) |
|  | 2 | 100  (96.8-100) | 97.7  (97.3-98.0) | 41.4  (35.8-47.3) | 100  (99.9-100) | 58.6  (52.2-64.4) | 97.7  (97.4-98.1) |
| Non-antimicrobial | 1 | 73.2  (72.2-74.3) | 100  (99.4-100) | 100  (99.9-100) | 24.9  (23.2-26.7) | 84.5  (84.2-84.9) | 75.4  (74.4-76.4) |
|  | 2 | 78.0  (77.0-79.0) | 100  (99.6-100) | 100  (99.9-100) | 42.1  (40.1-44.1) | 87.7  (87.3-88.0) | 81.0  (80.1-81.9) |

**Supplemental Table 3**. True positives (TPs), true negatives (TNs), false positives (FPs), and false negatives (FNs) for Claude Sonnet 4 by antimicrobial class: Phase 1 (Unguided) vs Phase 2 (Feedback-informed), n = 7,239 medication entries

|  | Phase | TPs (n, %) | TNs (n, %) | FPs (n, %) | FNs (n, %) |
| --- | --- | --- | --- | --- | --- |
| Antibiotic | 1 | 1,893 (26.2) | 5,311 (73.4) | 21 (0.3) | 14 (0.2) |
|  | 2 | 1,904 (26.3) | 5,310 (73.4) | 22 (0.3) | 3 (0) |
| Antifungal | 1 | 173 (2.4) | 7,056 (97.5) | 0 (0) | 10 (0.2) |
|  | 2 | 176 (2.4) | 7,055 (97.5) | 1 (0) | 7 (0.1) |
| Antiviral | 1 | 259 (3.6) | 6,959 (96.1) | 0 (0) | 21 (0.3) |
|  | 2 | 272 (3.8) | 6,959 (96.1) | 0 (0) | 8 (0.1) |
| Non-antimicrobial | 1 | 4,848 (66.9) | 2,325 (32.1) | 45 (0.6) | 21 (0.3) |
|  | 2 | 4,847 (67.0) | 2,353 (32.5) | 17 (0.2) | 22 (0.3) |

**Supplemental Table 4.** Performance classification metrics for Claude Sonnet 4 by antimicrobial class: Phase 1 (Unguided) vs Phase 2 (Feedback-informed)

| Class | Phase | Precision  (%, 95% CIs) | NPV  (%, 95% CIs) | Recall  (%, 95% CIs) | Specificity (%, 95% CIs) | F1 Score  (%, 95% CIs) | Accuracy  (%, 95% CIs) |
| --- | --- | --- | --- | --- | --- | --- | --- |
| Antibiotic | 1 | 98.9  (98.3-99.3) | 99.7  (99.6-99.8) | 99.3  (98.8-99.6) | 99.6  (99.4-99.7) | 99.1  (98.8-99.3) | 99.5  (99.3-99.7) |
|  | 2 | 98.9  (98.3-99.2) | 99.9  (99.8-100) | 99.8  (99.5-99.9) | 99.6  (99.4-99.7) | 99.3  (99.1-99.6) | 99.7  (99.5-99.8) |
| Antifungal | 1 | 100  (97.8-100) | 99.9  (99.7-99.9) | 94.5  (90.2-97.0) | 100  (99.9-100) | 97.2  (95.1-98.9) | 99.9  (99.7-99.9) |
|  | 2 | 99.4  (96.9-99.9) | 99.9  (99.8-100) | 96.2  (92.3-98.1) | 100  (99.9-100) | 97.8  (96.0-99.2) | 99.9  (99.8-99.9) |
| Antiviral | 1 | 100  (98.5-100) | 99.7  (99.5-99.8) | 92.5  (88.8-95.0) | 100  (99.9-100) | 96.1  (94.3-97.6) | 99.7  (99.6-99.8) |
|  | 2 | 100  (98.6-100) | 99.9  (99.8-99.9) | 97.1  (94.5-98.5) | 100  (99.9-100) | 98.6  (97.4-99.5) | 99.9  (99.8-99.9) |
| Non-antimicrobial | 1 | 99.1  (98.8-99.3) | 99.1  (98.6-99.4) | 99.6  (99.3-99.7) | 98.1  (97.5-98.6) | 99.3  (99.2-99.5) | 99.1  (98.8-99.3) |
|  | 2 | 99.7  (99.4-99.8) | 99.1  (98.6-99.4) | 99.5  (99.3-99.7) | 99.6  (98.9-99.6) | 99.6  (99.5-99.7) | 99.5  (99.3-99.6) |

**Supplemental Table 5**. True positives (TPs), true negatives (TNs), false positives (FPs), and false negatives (FNs) for Copilot by antimicrobial class: Phase 1 (Unguided) vs Phase 2 (Feedback-informed), n = 7,239 medication entries

|  | Phase | TPs (n, %) | TNs (n, %) | FPs (n, %) | FNs (n, %) |
| --- | --- | --- | --- | --- | --- |
| Antibiotic | 1 | 457 (6.3) | 5,332 (73.7) | 0 (0) | 1,450 (20) |
|  | 2 | 772 (10.7) | 5,332 (73.7) | 0 (0) | 1,135 (15.7) |
| Antifungal | 1 | 19 (0.3) | 7,056 (97.5) | 0 (0) | 164 (2.3) |
|  | 2 | 39 (0.5) | 7,056 (97.5) | 0 (0) | 144 (2) |
| Antiviral | 1 | 62 (0.9) | 6,959 (96.1) | 0 (0) | 218 (3) |
|  | 2 | 93 (1.3) | 6,959 (96.1) | 0 (0) | 187 (2.6) |
| Non-antimicrobial | 1 | 4,869 (67.3) | 538 (7.4) | 1,832 (25.3) | 0 (0) |
|  | 2 | 4,869 (67.3) | 904 (12.5) | 1,466 (20.3) | 0 (0) |

**Supplemental Table 6.** Performance classification metrics for Copilot by antimicrobial class: Phase 1 (Unguided) vs Phase 2 (Feedback-informed)

| Class | Phase | Precision  (%, 95% CIs) | NPV  (%, 95% CIs) | Recall  (%, 95% CIs) | Specificity (%, 95% CIs) | F1 Score  (%, 95% CIs) | Accuracy  (%, 95% CIs) |
| --- | --- | --- | --- | --- | --- | --- | --- |
| Antibiotic | 1 | 100  (99.2-100) | 78.6  (77.6-79.6) | 24.0  (22.125.9) | 100  (99.9-100) | 38.7  (36.2-41.3) | 80.0 (79.0-80.9) |
|  | 2 | 100  (99.5-100) | 82.4  (81.5-83.4) | 40.5  (38.3-42.7) | 100  (99.9-100) | 57.6  (55.4-59.9) | 84.3  (83.5-85.1) |
| Antifungal | 1 | 100  (83.2-100) | 97.7  (97.4-98.0) | 10.4  (6.7-15.6) | 100  (99.9-100) | 18.8  (11.3-26.5) | 97.7  (97.4-98.1) |
|  | 2 | 100  (91.1-100) | 98.0  (97.7-98.3) | 21.3  (16.0-27.8) | 100  (99.9-100) | 35.1  (26.5-42.9) | 98.0  (97.7-98.3) |
| Antiviral | 1 | 100  (94.2-100) | 97.0  (96.5-97.3) | 22.1  (17.7-27.4) | 100  (99.9-100) | 36.3  (29.8-42.7) | 97.0  (96.6-97.4) |
|  | 2 | 100  (96.0-100) | 97.4  (97.0-97.7) | 33.2  (28.0-38.9) | 100  (99.9-100) | 49.9  (43.6-56.0) | 97.4  (97.0-97.8) |
| Non-antimicrobial | 1 | 72.7  (71.6-73.7) | 100  (99.3-100) | 100  (99.9-100) | 22.7  (21.1-24.4) | 84.2  (83.9-84.5) | 74.7  (73.7-75.7) |
|  | 2 | 76.9  (75.8-77.9) | 100  (99.6-100) | 100  (99.9-100) | 38.1  (36.2-40.1) | 86.9  (86.6-87.3) | 79.7  (78.8-80.7) |

**Supplemental Table 7**. True positives (TPs), true negatives (TNs), false positives (FPs), and false negatives (FNs) for Gemini by antimicrobial class: Phase 1 (Unguided) vs Phase 2 (Feedback-informed), n = 7,239 medication entries

|  | Phase | TPs (n, %) | TNs (n, %) | FPs (n, %) | FNs (n, %) |
| --- | --- | --- | --- | --- | --- |
| Antibiotic | 1 | 1,836 (25.4) | 5,326 (73.6) | 6 (0.1) | 71 (1) |
|  | 2 | 1,905 (26.3) | 5,320 (73.5) | 12 (0.2) | 2 (0) |
| Antifungal | 1 | 181 (2.5) | 7,050 (97.4) | 6 (0.1) | 2 (0) |
|  | 2 | 183 (2.5) | 7,049 (97.4) | 7 (0.1) | 0 (0) |
| Antiviral | 1 | 279 (3.9) | 6,948 (96.0) | 11 (0.2) | 1 (0) |
|  | 2 | 280 (3.9) | 6,949 (96.0) | 10 (0.1) | 0 (0) |
| Non-antimicrobial | 1 | 4,847 (67) | 2,297 (31.7) | 73 (1) | 22 (0.3) |
|  | 2 | 4,842 (66.9) | 2,370 (32.7) | 0 (0) | 27 (0.4) |

**Supplemental Table 8.** Performance classification metrics for Gemini by antimicrobial class: Phase 1 (Unguided) vs Phase 2 (Feedback-informed)

| Class | Phase | Precision  (%, 95% CIs) | NPV  (%, 95% CIs) | Recall  (%, 95% CIs) | Specificity (%, 95% CIs) | F1 Score  (%, 95% CIs) | Accuracy  (%, 95% CIs) |
| --- | --- | --- | --- | --- | --- | --- | --- |
| Antibiotic | 1 | 99.7  (99.3-99.9) | 98.7  (98.3-99.0) | 96.3  (95.3-97.0) | 99.9  (99.8-99.9) | 97.9  (97.5-98.4) | 98.9  (98.7-99.1) |
|  | 2 | 99.4  (98.9-99.6) | 100  (99.9-100) | 99.9  (99.6-100) | 99.8  (99.6-99.9) | 99.6  (99.4-99.8) | 99.8  (99.7-99.9) |
| Antifungal | 1 | 96.8  (93.2-98.5) | 100  (99.9-100) | 98.9  (96.1-99.7) | 99.9  (99.8-100) | 97.8  (96.2-99.2) | 99.9  (99.8-99.9) |
|  | 2 | 96.3  (92.6-98.2) | 100  (99.9-100) | 100  (97.9-100) | 99.9  (99.8-100) | 98.1  (96.6-99.5) | 99.9  (99.8-100) |
| Antiviral | 1 | 96.2  (93.3-97.9) | 100  (99.9-100) | 99.6  (98.0-99.9) | 99.8  (99.7-99.9) | 97.9  (96.7-98.9) | 99.8  (99.7-99.9) |
|  | 2 | 96.6  (93.8-98.1) | 100  (99.9-100) | 100  (98.6-100) | 99.9  (99.7-99.9) | 98.2  (97.1-99.3) | 99.9  (99.7-99.9) |
| Non-antimicrobial | 1 | 98.5  (98.1-98.8) | 99.1  (98.6-99.4) | 99.5  (99.3-99.7) | 96.9  (96.1-97.5) | 99.0  (98.8-99.2) | 98.7  (98.4-98.9) |
|  | 2 | 100  (99.9-100) | 98.9  (98.4-99.2) | 99.4  (99.2-99.6) | 100  (99.8-100) | 99.7  (99.6-99.8) | 99.6  (99.5-99.7) |

**Supplemental Table 9.** Confusion matrix of LLMs in antimicrobial classification: Phase 2 (Feedback-informed)

1. ChatGPT-3.5

|  | Predicted Antibiotic | Predicted Antifungal | Predicted Antiviral | Predicted Non-antimicrobial | Total |
| --- | --- | --- | --- | --- | --- |
| Actual Antibiotic | 787 | 1 | - | 1,119 | 1,907 |
| Actual Antifungal | - | 94 | - | 89 | 183 |
| Actual Antiviral | - | - | 116 | 164 | 280 |
| Actual Non-antimicrobial | - | - | - | 4,869 | 4,869 |

1. Claude Sonnet 4

|  | Predicted Antibiotic | Predicted Antifungal | Predicted Antiviral | Predicted Non-antimicrobial | Total |
| --- | --- | --- | --- | --- | --- |
| Actual Antibiotic | 1,904 | 1 | - | 2 | 1,907 |
| Actual Antifungal | - | 176 | - | 7 | 183 |
| Actual Antiviral | - | - | 272 | 8 | 280 |
| Actual Non-antimicrobial | 22 | - | - | 4,847 | 4,869 |

1. Copilot

|  | Predicted Antibiotic | Predicted Antifungal | Predicted Antiviral | Predicted Non-antimicrobial | Total |
| --- | --- | --- | --- | --- | --- |
| Actual Antibiotic | 772 | - | - | 1,135 | 1,907 |
| Actual Antifungal | - | 39 | - | 144 | 183 |
| Actual Antiviral | - | - | 93 | 187 | 280 |
| Actual Non-antimicrobial | - | - | - | 4,869 | 4,869 |
